# Data supporting midpoint-weighting life cycle assessment and energy forms of cumulative exergy demand for horticultural crops

**DOI:** 10.1016/j.dib.2020.106490

**Published:** 2020-11-04

**Authors:** Fatemeh Mostashari-Rad, Hassan Ghasemi-Mobtaker, Morteza Taki, Mohammad Ghahderijani, Zahra Saber, Kwok-Wing Chau, Ashkan Nabavi-Pelesaraei

**Affiliations:** a Department of Agricultural Biotechnology, Faculty of Agricultural Sciences, University of Guilan, Rasht, Iran; b Department of Agricultural Machinery Engineering, Faculty of Agricultural Engineering and Technology, University of Tehran, Karaj, Iran; c Department of Agricultural Machinery and Mechanization, Agricultural Sciences and Natural Resources University of Khuzestan, Mollasani, Iran; d Department of Agricultural Systems Engineering, Science and Research Branch, Islamic Azad University, Tehran, Iran; e Department of Agronomy and Plant Breeding, Sari Agricultural Sciences and Natural Resources University, Sari, Iran; f Department of Civil and Environmental Engineering, Hong Kong Polytechnic University, Hung Hom, Kowloon, Hong Kong; g Head of Process Engineering & Systems Improvement, Management of Fruit and Vegetables Organizations, Tehran Municipality, Tehran, Iran

**Keywords:** Life cycle assessment, Cumulative exergy demand, Horticultural crop, Environmental midpoint, Recipe2016, Weighting

## Abstract

With an increasing demand of horticultural crops, it is critical to examine environmental damages and exergy impacts and evaluate their potential in producing sustainable products of agricultural systems. As such, environmental midpoints of five dominated horticultural products, namely, hazelnut, watermelon, tea, kiwifruit, and citrus, are scrutinized using life cycle assessment approach in Guilan province, Iran. Each crop is considered under a separate scenario and 10 tons of yield is determined as the functional unit. ReCiPe2016, as a new approach, is used for computation of 17 midpoints. Moreover, a weighting analysis is undertaken to find the share of each input in environmental damages with dimensionless notation. In the second part of this paper, cumulative exergy demand (CExD) is applied for evaluation of energy forms in each scenario. Data are presented under two sectors in the main article. The first part is midpoint results of each crop and the second part depicts energy forms of CExD with input rate in each category. Besides, the supplementary files contain raw material of each input, midpoint physical rate, share of each input to contribute midpoint, raw data of weighted damages and share of each input in total weighted damages.

## Specifications Table

**Table:** 

Subject	Environmental Science
Specific subject area	Life cycle assessment (LCA) and cumulative exergy demand (CExD)
Type of data	Table and chart
How data were acquired	Initial data are taken face-to-face from orchardists in Guilan province of Iran and are subsequently converted into exergoenvironmental damages using the “ReCiPe2016” and “CExD” methods available in EcoInvent®3.6 database with SimaPro 9.0.0 software and PestLCI 2.0 database with Analytica 5.0 software.
Data format	Raw and analyzed
Parameters for data collection	Horticultural input energy and materials along with their emissions to soil, air, and water in watermelon, kiwifruit, tea, hazelnut, and citrus production.
Description of data collection	Physical amounts of agricultural inputs with their midpoint emissions and weighted score of endpoints are considered as raw data for midpoint-weighting LCA. Moreover, energy forms of each agricultural input are considered as raw data in CExD analysis.
Data source location	Guilan province of Iran
Data accessibility	With the article
Related research article	F. Mostashari-Rad, H. Ghasemi-Mobtaker, M. Taki, M. Ghahderijani, A. Kaab, K. Chau, A. Nabavi-Pelesaraei, Exergoenvironmental damages assessment of horticultural crops using ReCiPe2016 and cumulative exergy demand frameworks, J. Clean. Prod. 278 (2021) 123,788.
	https://doi.org/10.1016/j.jclepro.2020.123788

## Data Value

• The data provide an applicable database to perform LCA of agricultural production (in particular, environmental LCA midpoints can be computed for horticultural crops).
• These data provide an applicable database to perform exergoenvironmental energy forms of agricultural production (more specifically, CExD energy forms can be computed for horticultural crops).
• These data can be directly used to perform LCA and exergy demand of agri-food post-harvesting systems such as production of jams and compotes (more specifically, analyzed data can be entered as inventory data during agricultural processing production life cycle).
• These data can offer a critical approach for the determination of the importance of exergoenvironmental damages in agricultural production, and especially weighting analysis of horticultural crops.
• The data can be of use to academics and researchers using life cycle cost analysis of agricultural production (more specifically, social cost of emissions can be obtained from environmental damages in horticultural production life cycle).

## 1 Data Description

LCA details for horticultural crops in Guilan province are presented in this data article. Moreover, details of energy form analysis computed by CExD method are also offered in this article. In fact, the data provided are supplementary information related to our recent published work [1].

The first part of the supplementary materials, which is designated as “Raw data of inputs for horticultural crops.xlsx”, presents the input physical amount of each farm for five investigated horticultural crops based on foreground data.

Table 1 presents the midpoint damages of environmental LCA following the ReCiPe2016 method for 5 dominated horticultural crops in Guilan province, Iran, namely, watermelon, hazelnut, tea, kiwifruit, and citrus. In fact, Table 1 lists 17 midpoints of ReCiPe2016 and their relationships with endpoints are illustrated in Mostashari-Rad et al. [1]. The physical amount of each midpoint is specified based on the mentioned crops.

**Table 1:** Total midpoint data of ReCiPe2016 for production of 10 t horticultural crops in Guilan province, Iran.

		Horticultural crops				
Midpoint	Unit	Citrus	Hazelnut	Kiwifruit	Tea	Watermelon
Global warming	kg CO_2_	2060.71	16,040.48	3197.85	10,656.15	3404.22
Stratos. ozone depletion	kg CFC11	0.03	0.20	0.05	0.15	0.07
Ionizing radiation	kBq Co-60	89.53	668.54	97.46	207.58	117.10
Trop. ozone formation (hum)	kg NOx	5.28	31.45	6.65	11.04	6.29
Particulate matter	kg PM2.5	5.48	41.10	7.79	16.35	8.20
Trop. ozone (eco)	kg NOx	5.36	32.22	6.74	11.18	6.39
Terrestrial acidification	kg SO_2_	31.06	228.04	46.69	104.83	49.91
Freshwater eutrophication	kg P	0.76	6.60	0.91	1.41	0.89
Terrestrial ecotoxicity	kg 1,4-DCB	8208.28	303,736.60	10,531.82	19,099.74	9731.06
Freshwater ecotoxicity	kg 1,4-DCB	306.58	19,079.19	356.09	440.17	201.01
Marine ecotoxicity	kg 1,4-DCB	103.71	4008.24	132.81	224.04	115.36
Human toxicity (cancer)	kg 1,4-DCB	34.63	343.24	47.65	97	59.11
Human toxicity (non-cancer)	kg 1,4-DCB	1326.48	12,291.83	1863.20	4156.24	2173.48
Land use	m2a crop	349.95	1882.77	288.97	582.19	348.70
Mineral resource	kg Cu	10.43	115.22	14.43	29.91	17.39
Fossil resources	kg oil	262.01	2150.22	359.16	647.27	381.07
Water use	m^3^	37.87	216.59	39.15	88	48.43

There are five parts of the supplementary materials in this data paper and the first part is mentioned above. The second part of the supplementary materials, which is designated as “Raw data of midpoints for horticultural crops.docx”, presents the midpoint details of each horticultural crop based on each consumed input in the production process (Table S1 to Table S5). These tables are also used to constitute the third part of the supplementary materials, which is designated as “Midpoints figures of horticultural crops.docx”. It displays the share of each input to participate to 17 midpoints for citrus (Figure S1 based on Table S1 raw data), hazelnut (Figure S2 based on Table S2 raw data), kiwifruit (Figure S3 based on Table S3 raw data), tea (Figure S4 based on Table S4 raw data) and watermelon (Figure S5 based on Table S5 raw data).

The fourth part of the supplementary materials, which is designated as “Raw data of weighting analysis.xlsx”, displays the raw data of weighted score for ReCiPe2016 endpoints. These data are used to perform the last part of the supplementary materials, which is designated as “Weighting figures of horticultural crops.docx”. It displays the share of each input in environmental endpoints damage for citrus (Figure S6), hazelnut (Figure S7), kiwifruit (Figure S8), tea (Figure S9) and watermelon (Figure S10).

Finally, Table 2 illustrates the amounts of six energy forms of CExD method, which are described by Mostashari-Rad et al. [1]. It should be noted that each input's energy consumption is considered as the basis for CExD analysis in Table 2.

**Table 2:** Details of energy form data for production of 10 t horticultural crops in Guilan province, based on breakdown of energy consumer factors.

			Energy consumer factors									
Crop	Energy form	Unit	Agricultural machinery	Lubricating oil	Nitrogen	Phosphate	Potassium	FYM	Pesticides	Fungicides	Diesel fuel	Electricity
Citrus	Non-renewable, fossil	MJ	523.84	126.63	4592.86	1415.81	1730.63	0	210.13	201.56	2340.22	800.74
	Renewable, kinetic	MJ	3.45	0.18	17.55	9.81	8.73	0	1.29	1.98	0.83	0.72
	Renewable, potential	MJ	25.66	1.13	98.61	60.66	56.99	0	6.51	10.10	4.29	16.98
	Non-renewable, primary	MJ	0.39	1.36E-03	65.25	9.98	248.40	0	0.01	0	0.01	4.03E-03
	Non-renewable, metals	MJ	49.38	0.78	249.50	60.69	36.19	0	3.82	0.13	1.43	2.12
	Non-renewable, minerals	MJ	2.45	0.30	127.63	297.82	26.80	0	3.18	0.08	1.29	0.33

Hazelnut	Non-renewable, fossil	MJ	12,131.84	2853.67	23,958.51	13,375.09	7654.12	0	14,897.47	14,357.22	10,430.26	–
	Renewable, kinetic	MJ	79.90	4.06	91.53	92.69	38.62	0	91.54	140.87	3.68	–
	Renewable, potential	MJ	594.37	25.54	514.39	573.09	252.06	0	461.34	719.48	19.10	–
	Non-renewable, primary	MJ	9.05	0.03	340.36	94.31	1098.59	0	0.48	0	0.03	–
	Non-renewable, metals	MJ	1143.63	17.49	1301.52	573.37	160.07	0	270.58	8.94	6.38	–
	Non-renewable, minerals	MJ	56.67	6.66	665.76	2813.53	118.55	0	225.13	5.99	5.73	–

Kiwifruit	Non-renewable, fossil	MJ	855.72	153.39	8249.10	1540.35	606.44	0	234.49	226.38	2478.03	1945.12
	Renewable, kinetic	MJ	5.64	0.22	31.51	10.67	3.06	0	1.44	2.22	0.87	1.75
	Renewable, potential	MJ	41.92	1.37	177.11	66	19.97	0	7.26	11.34	4.54	41.25
	Non-renewable, primary	MJ	0.64	1.64E-03	117.19	10.86	87.04	0	0.01	0	0.01	0.01
	Non-renewable, metals	MJ	80.67	0.94	448.13	66.03	12.68	0	4.26	0.14	1.52	5.16
	Non-renewable, minerals	MJ	4	0.36	229.23	324.02	9.39	0	3.54	0.09	1.36	0.80

Tea	Non-renewable, fossil	MJ	684.87	147.23	24,573.20	2003.97	–	249.04	241.82	1345.38	249.04	–
	Renewable, kinetic	MJ	4.51	0.21	93.88	13.89	–	1.53	2.37	0.47	1.53	–
	Renewable, potential	MJ	33.55	1.32	527.59	85.87	–	7.71	12.12	2.46	7.71	–
	Non-renewable, primary	MJ	0.51	1.58E-03	349.09	14.13	–	0.01	0	4.09E-03	0.01	–
	Non-renewable, metals	MJ	64.56	0.90	1334.92	85.91	–	4.52	0.15	0.82	4.52	–
	Non-renewable, minerals	MJ	3.20	0.34	682.84	421.55	–	3.76	0.10	0.74	3.76	–

Watermelon	Non-renewable, fossil	MJ	1107.65	163.75	11,345.39	1462.64	546.82	0	108.90	104.55	1148.93	1249.97
	Renewable, kinetic	MJ	7.30	0.23	43.34	10.14	2.76	0	0.67	1.03	0.41	1.12
	Renewable, potential	MJ	54.27	1.47	243.59	62.67	18.01	0	3.37	5.24	2.10	26.51
	Non-renewable, primary	MJ	0.83	1.75E-03	161.17	10.31	78.48	0	3.50E-03	0	3.49E-03	0.01
	Non-renewable, metals	MJ	104.42	1	616.33	62.70	11.44	0	1.98	0.07	0.70	3.31
	Non-renewable, minerals	MJ	5.17	0.38	315.27	307.67	8.47	0	1.65	0.04	0.63	0.51

## 2 Experimental Design, Materials and Methods

Environmental damages caused by the agricultural sector has become increasingly apparent due to the growing population and the consequent increase in horticultural production throughout the world [2]. Accordingly, one of the most important debates around horticultural production has focused on analyzing environmental damage assessment [3]. LCA is a method used to measure the environmental burden of a process or product along with the assessment of inputs, outputs, and their pertinent emissions. This method supports enhanced environmental management and is an integral tool for evaluating environmental burdens of many agricultural products’ supply chains [2]. It can also be used to compare environmental effects of different options and to determine the optimal option [4]. In fact, LCA is a cradle-to-grave approach to crop management, which begins with the initial collection of raw materials from the earth and continues to the point where the residuals returns to the earth [5]. It is also a powerful tool to reduce social consequences, increase economic stability and enhance resource efficiency [6]. High energy consumption in agricultural operations, along with the production of various environmental pollutants, has a risky impact on the environment. CExD analysis is another analytical instrument applied in environmental researches. In fact, this method addresses the entire exergy content required for a product or service [7,8]. Based on Peters et al. [9], a goal of CExD analysis is to attain competency of a system or procedure by reducing damages or lack in exergy.

Although the endpoint analysis of LCA and energy forms of CExD can offer an acceptable concept for selecting an exergoenvironmental-friendly production system, the presentation of more details especially in midpoints, weightings and energy form levels can be effective for future studies intending to follow exergoenvironmental aspects in other horticultural crop cultivation and agricultural processing production.

Accordingly, the following steps are considered for exergoenvironmental analysis of horticultural products in Guilan province:

1 Defining 5 scenarios for horticultural crops, namely, kiwifruit, watermelon, tea, hazelnut, and citrus, with their inventory expressed in Ref. [1].
2 Analysis of total endpoints and total CExD, which is expressed in Ref. [1].
3 Midpoint analysis of each scenario based on the physical amount and disturbing framework offered in this data paper.
4 Weighting analysis of each scenario based on contributed survey of inputs to environmental damages.
5 Offering details of energy forms with CExD framework for each scenario based on energy consumer inputs.

LCA is performed in 4 different steps, including determining the scope and goals, analyzing the life cycle inventory (LCI), assessing the impact of the life cycle (LCIA), and finally interpreting results of the life cycle [10,11]:

### 2.1 Scope and goals definition

The first step in LCA is to define the goals and scope [12], in which determining the functional unit and the boundary of the system are essential steps [13]. In this research work, five horticultural crops, namely, hazelnut, watermelon, tea, kiwifruit, and citrus, are considered for LCA. The boundary of the system involves the operations of the entire orchard and the use of all inputs for horticultural products under all scenarios. Besides, the functional unit is set as 10 tons (t) of the yield of the mentioned crops.

### 2.2 LCI analysis

The analysis of inventory is the second stage of LCA, involving the computation of the amount of each output and input [14]. Based on the functional unit, this stage denotes a comprehensive collection of energy outputs, inputs, materials, along with the emissions to soil, air, and water in all steps of the system [15].

The inventory data consists of two sectors, namely, Off-Orchard and On-Orchard emissions. Off-Orchard emissions are acquired from the information of foreground system involving amounts and types of materials as well as direct use of energy in horticultural crop production, which are demonstrated in the “Raw data of inputs for horticultural crops.xlsx” of the supplementary materials. On-Orchard emissions are associated with the information of background system involving direct emissions of diesel fuel combustion in agricultural machinery, emissions to air owing to human labor activities, emissions to soil, water, and air owing to fertilizers, which are taken from EcoInvent®3.6. Emissions derived from biocides are taken from PestLCI 2.0 model in Analytica software.

### 2.3 LCIA

LCIA translates LCI into damages [16], which helps interpreting the assessment via converting all emissions and resources into scores of environmental impacts [17]. There are various tools for LCIA including CML2 baseline 2000, IMPACT2002+, ReCiPe2008, USEtox, ReCiPe2016, etc. The adoption of environmental categories depends on the applied LCIA tool. In this study, ReCiPe2016, as an up-to-date approach in damages assessment [4], is considered for LCIA and the analysis is computed by SimaPro V.9.0.0.

The environmental impacts in the endpoint level of ReCiPe2016 are gathered into three damage types: ecosystems, resources, and human health [18]. Although results are easier interpreted and taken into account by decision makers in endpoint level, its drawback is a higher statistical uncertainty [19]. Accordingly, more details of the results are essential for making decision, which is more accurate in the analysis by midpoint level analysis. ReCiPe2016 consists of 17 categories in midpoint level as mentioned above. Total physical amount of each midpoint for each horticultural crop midpoint is illustrated in Table 1. For offering more details about midpoints, “Midpoints tables of horticultural crops.docx” and “Midpoints figures of horticultural crops.docx” are demonstrated in the supplementary materials, which show the physical amount of each input and its share to midpoints, respectively.

### 2.4 Weighting

Weighting methodology is considered as an analysis mechanism in identifying the major dimension and issues to assess. Weighting helps a researcher to represent results of the research after the characterization stage by applying a common reference impact [20]. Two parts of the supplementary materials, namely, “Raw data of weighting analysis.xlsx” and “Weighting figures of horticultural crops.docx”, show the weighting score and their share to constitute each weighted endpoint, respectively.

### 2.5 CExD

As an innovative approach, CExD evaluates various problems of energy use in the process of crop production. Some parameters can be acutely addressed by CExD analysis such as renewability indicator computations and cumulative degree of perfect [21]. Finally, six energy forms of CExD analysis, namely, Renewable, kinetic, Non-renewable, fossil, Renewable, potential, Non-renewable, minerals, Non-renewable, primary, and Non-renewable, metals, are surveyed in this data paper based on separated inputs (Table 2).

## Declaration of Competing Interest

The authors declare that they have no known competing financial interests or personal relationships that could have appeared to influence the work reported in this paper.

## Appendix Supplementary materials

Image, application 1

Image, application 2

Image, application 3

Image, application 4

Image, application 5
