# Supplementary material for: Data supporting midpoint-weighting life cycle assessment and energy forms of cumulative exergy demand for horticultural crops
Source: Data Brief. 2020 Nov 4;33:106490. doi: 10.1016/j.dib.2020.106490 (PMC7658572; doi:10.1016/j.dib.2020.106490)
Supplement: Supplementary file 2 [file mmc2.docx]

| **Table S1**  Midpoints data of ReCiPe2016 for producing 10 t of citrus in Guilan province of Iran based on the breakdown of emissions factors. | | | | | | | | | | | | |
| --- | --- | --- | --- | --- | --- | --- | --- | --- | --- | --- | --- | --- |
| Midpoint | Unit | Emissions factors | | | | | | | | | | |
|  |  | On-Orchard emissions | Agricultural machinery | Lubricating oil | Nitrogen | Phosphate | Potassium | FYM | Pesticides | Fungicides | Diesel fuel | Electricity |
| Global warming | kg CO_2_ | 747.26 | 53.18 | 2.72 | 768.53 | 95.73 | 289.01 | 0 | 13.26 | 16.59 | 22.25 | 52.18 |
| Stratos. ozone depletion | kg CFC11 | 0.02 | 1.54E-05 | 2.10E-06 | 0.01 | 1.32E-04 | 2.24E-03 | 0 | 4.98E-05 | 8.99E-06 | 3.68E-05 | 2.71E-05 |
| Ionizing radiation | kBq Co-60 | 0 | 2.45 | 0.17 | 35.04 | 9.13 | 37.54 | 0 | 0.92 | 1.41 | 1.72 | 1.15 |
| Trop. ozone formation (hum) | kg NOx | 2.57 | 0.12 | 0.01 | 1.45 | 0.29 | 0.61 | 0 | 0.04 | 1.01E-03 | 0.08 | 0.09 |
| Particulate matter | kg PM2.5 | 3.39 | 0.10 | 0.01 | 0.91 | 0.36 | 0.54 | 0 | 0.04 | 0.02 | 0.06 | 0.06 |
| Trop. ozone (eco) | kg NOx | 2.59 | 0.13 | 0.02 | 1.47 | 0.30 | 0.63 | 0 | 0.04 | 1.19E-03 | 0.08 | 0.10 |
| Terrestrial acidification | kg SO_2_ | 23.82 | 0.18 | 0.01 | 3.42 | 0.85 | 2.25 | 0 | 0.10 | 0.07 | 0.19 | 0.17 |
| Freshwater eutrophication | kg P | 0.41 | 0.03 | 8.45E-04 | 0.14 | 0.10 | 0.07 | 0 | 7.71E-03 | 2.52E-04 | 2.62E-03 | 2.57E-03 |
| Terrestrial ecotoxicity | kg 1,4-DCB | 3946.64 | 231.78 | 7.50 | 2465.64 | 635.99 | 729.01 | 0 | 50.76 | 4.55 | 29.75 | 106.65 |
| Freshwater ecotoxicity | kg 1,4-DCB | 270.30 | 1.84 | 0.07 | 19.20 | 6.19 | 6.48 | 0 | 0.58 | 0.02 | 0.21 | 1.70 |
| Marine ecotoxicity | kg 1,4-DCB | 54.60 | 2.63 | 0.10 | 27 | 8.77 | 7.45 | 0 | 0.60 | 0.02 | 0.36 | 2.16 |
| Human toxicity (cancer) | kg 1,4-DCB | 0.29 | 6.21 | 0.09 | 15 | 5.12 | 6.44 | 0 | 0.50 | 0.02 | 0.47 | 0.49 |
| Human toxicity (non-cancer) | kg 1,4-DCB | 199.14 | 55.18 | 2.21 | 649.13 | 213.11 | 175.36 | 0 | 13.47 | 0.86 | 6.34 | 11.68 |
| Land use | m2a crop | 0 | 1.07 | 0.05 | 103.02 | 19.04 | 224.37 | 0 | 0.26 | 1.79 | 0.27 | 0.09 |
| Mineral resource | kg Cu | 0 | 1.24 | 0.01 | 4.24 | 3.61 | 0.89 | 0 | 0.36 | 2.04E-03 | 0.04 | 0.03 |
| Fossil resources | kg oil | 0 | 11.28 | 2.75 | 102.05 | 31.14 | 37.64 | 0 | 4.63 | 3.94 | 50.45 | 18.12 |
| Water use | m^3^ | 0 | 0.31 | 0.03 | 15.28 | 3.87 | 17.86 | 0 | 0.05 | 0.13 | 0.25 | 0.10 |

| **Table S2**  Midpoints data of ReCiPe2016 for producing 10 t of hazelnut in Guilan province of Iran based on the breakdown of emissions factors. | | | | | | | | | | | |
| --- | --- | --- | --- | --- | --- | --- | --- | --- | --- | --- | --- |
| Midpoint | Unit | Emissions factors | | | | | | | | | |
|  |  | On-Orchard emissions | Agricultural machinery | Lubricating oil | Nitrogen | Phosphate | Potassium | FYM | Pesticides | Fungicides | Diesel fuel |
| Global warming | kg CO_2_ | 6334.79 | 1231.64 | 61.20 | 4009 | 904.36 | 1278.22 | 0 | 940.45 | 1181.65 | 99.18 |
| Stratos. ozone depletion | kg CFC11 | 0.10 | 3.57E-04 | 4.74E-05 | 0.08 | 1.24E-03 | 0.01 | 0 | 3.53E-03 | 6.41E-04 | 1.64E-04 |
| Ionizing radiation | kBq Co-60 | 0 | 56.65 | 3.84 | 182.80 | 86.30 | 166.02 | 0 | 65.09 | 100.16 | 7.69 |
| Trop. ozone formation (hum) | kg NOx | 12.34 | 2.78 | 0.32 | 7.57 | 2.78 | 2.72 | 0 | 2.50 | 0.07 | 0.35 |
| Particulate matter | kg PM2.5 | 23.59 | 2.38 | 0.13 | 4.74 | 3.40 | 2.38 | 0 | 2.64 | 1.56 | 0.28 |
| Trop. ozone (eco) | kg NOx | 12.41 | 3.06 | 0.43 | 7.66 | 2.83 | 2.79 | 0 | 2.57 | 0.09 | 0.38 |
| Terrestrial acidification | kg SO_2_ | 174.89 | 4.26 | 0.31 | 17.83 | 8.07 | 9.96 | 0 | 6.90 | 5 | 0.83 |
| Freshwater eutrophication | kg P | 3.45 | 0.59 | 0.02 | 0.75 | 0.94 | 0.29 | 0 | 0.55 | 0.02 | 0.01 |
| Terrestrial ecotoxicity | kg 1,4-DCB | 272049.99 | 5367.98 | 168.92 | 12861.96 | 6008.18 | 3224.24 | 0 | 3598.65 | 324.09 | 132.60 |
| Freshwater ecotoxicity | kg 1,4-DCB | 18804.50 | 42.70 | 1.58 | 100.17 | 58.45 | 28.65 | 0 | 40.97 | 1.25 | 0.91 |
| Marine ecotoxicity | kg 1,4-DCB | 3642.40 | 60.98 | 2.31 | 140.85 | 82.89 | 32.97 | 0 | 42.69 | 1.54 | 1.60 |
| Human toxicity (cancer) | kg 1,4-DCB | 3.10 | 143.90 | 2.05 | 78.27 | 48.33 | 28.48 | 0 | 35.47 | 1.57 | 2.08 |
| Human toxicity (non-cancer) | kg 1,4-DCB | 3744.63 | 1278.01 | 49.73 | 3386.19 | 2013.27 | 775.55 | 0 | 955.02 | 61.18 | 28.26 |
| Land use | m2a crop | 0 | 24.75 | 1.10 | 537.39 | 179.84 | 992.31 | 0 | 18.53 | 127.65 | 1.20 |
| Mineral resource | kg Cu | 0 | 28.71 | 0.21 | 22.14 | 34.13 | 3.92 | 0 | 25.78 | 0.14 | 0.17 |
| Fossil resources | kg oil | 0 | 261.34 | 61.86 | 532.36 | 294.21 | 166.45 | 0 | 328.15 | 280.97 | 224.86 |
| Water use | m^3^ | 0 | 7.12 | 0.64 | 79.69 | 36.57 | 79 | 0 | 3.56 | 8.93 | 1.10 |

| **Table S3**  Midpoints data of ReCiPe2016 for producing 10 t of kiwifruit in Guilan province of Iran based on the breakdown of emissions factors. | | | | | | | | | | | | |
| --- | --- | --- | --- | --- | --- | --- | --- | --- | --- | --- | --- | --- |
| Midpoint | Unit | Emissions factors | | | | | | | | | | |
|  |  | On-Orchard emissions | Agricultural machinery | Lubricating oil | Nitrogen | Phosphate | Potassium | FYM | Pesticides | Fungicides | Diesel fuel | Electricity |
| Global warming | kg CO_2_ | 1338.18 | 86.87 | 3.29 | 1380.33 | 104.15 | 101.27 | 0 | 14.80 | 18.63 | 23.56 | 126.75 |
| Stratos. ozone depletion | kg CFC11 | 0.03 | 2.52E-05 | 2.55E-06 | 0.03 | 1.43E-04 | 7.84E-04 | 0 | 5.55E-05 | 1.01E-05 | 3.90E-05 | 6.58E-05 |
| Ionizing radiation | kBq Co-60 | 0 | 4 | 0.21 | 62.94 | 9.94 | 13.15 | 0 | 1.02 | 1.58 | 1.83 | 2.79 |
| Trop. ozone formation (hum) | kg NOx | 2.94 | 0.20 | 0.02 | 2.61 | 0.32 | 0.22 | 0 | 0.04 | 1.13E-03 | 0.08 | 0.23 |
| Particulate matter | kg PM2.5 | 5.13 | 0.17 | 0.01 | 1.63 | 0.39 | 0.19 | 0 | 0.04 | 0.02 | 0.07 | 0.14 |
| Trop. ozone (eco) | kg NOx | 2.95 | 0.22 | 0.02 | 2.64 | 0.33 | 0.22 | 0 | 0.04 | 1.34E-03 | 0.09 | 0.23 |
| Terrestrial acidification | kg SO_2_ | 37.73 | 0.30 | 0.02 | 6.14 | 0.93 | 0.79 | 0 | 0.11 | 0.08 | 0.20 | 0.40 |
| Freshwater eutrophication | kg P | 0.46 | 0.04 | 1.02E-03 | 0.26 | 0.11 | 0.02 | 0 | 0.01 | 2.83E-04 | 2.77E-03 | 0.01 |
| Terrestrial ecotoxicity | kg 1,4-DCB | 4415.91 | 378.63 | 9.08 | 4428.47 | 691.94 | 255.46 | 0 | 56.64 | 5.11 | 31.50 | 259.07 |
| Freshwater ecotoxicity | kg 1,4-DCB | 304.50 | 3.01 | 0.09 | 34.49 | 6.73 | 2.27 | 0 | 0.64 | 0.02 | 0.22 | 4.12 |
| Marine ecotoxicity | kg 1,4-DCB | 61.41 | 4.30 | 0.12 | 48.50 | 9.55 | 2.61 | 0 | 0.67 | 0.02 | 0.38 | 5.24 |
| Human toxicity (cancer) | kg 1,4-DCB | 0.36 | 10.15 | 0.11 | 26.95 | 5.57 | 2.26 | 0 | 0.56 | 0.02 | 0.49 | 1.18 |
| Human toxicity (non-cancer) | kg 1,4-DCB | 260.11 | 90.14 | 2.67 | 1165.89 | 231.86 | 61.45 | 0 | 15.03 | 0.96 | 6.71 | 28.37 |
| Land use | m2a crop | 0 | 1.75 | 0.06 | 185.03 | 20.71 | 78.62 | 0 | 0.29 | 2.01 | 0.29 | 0.22 |
| Mineral resource | kg Cu | 0 | 2.03 | 0.01 | 7.62 | 3.93 | 0.31 | 0 | 0.41 | 2.29E-03 | 0.04 | 0.08 |
| Fossil resources | kg oil | 0 | 18.43 | 3.33 | 183.30 | 33.88 | 13.19 | 0 | 5.17 | 4.43 | 53.42 | 44.01 |
| Water use | m^3^ | 0 | 0.50 | 0.03 | 27.44 | 4.21 | 6.26 | 0 | 0.06 | 0.14 | 0.26 | 0.24 |

| **Table S4**  Midpoints data of ReCiPe2016 for producing 10 t of tea in Guilan province of Iran based on the breakdown of emissions factors. | | | | | | | | | | |
| --- | --- | --- | --- | --- | --- | --- | --- | --- | --- | --- |
| Midpoint | Unit | Emissions factors | | | | | | | | |
|  |  | On-Orchard emissions | Agricultural machinery | Lubricating oil | Nitrogen | Phosphate | FYM | Pesticides | Fungicides | Diesel fuel |
| Global warming | kg CO_2_ | 6287.69 | 69.53 | 3.16 | 4111.86 | 135.50 | 0 | 15.72 | 19.90 | 12.79 |
| Stratos. ozone depletion | kg CFC11 | 0.07 | 2.01E-05 | 2.44E-06 | 0.08 | 1.86E-04 | 0 | 5.90E-05 | 1.08E-05 | 2.12E-05 |
| Ionizing radiation | kBq Co-60 | 0 | 3.20 | 0.20 | 187.49 | 12.93 | 0 | 1.09 | 1.69 | 0.99 |
| Trop. ozone formation (hum) | kg NOx | 2.60 | 0.16 | 0.02 | 7.76 | 0.42 | 0 | 0.04 | 1.21E-03 | 0.05 |
| Particulate matter | kg PM2.5 | 10.73 | 0.13 | 0.01 | 4.86 | 0.51 | 0 | 0.04 | 0.03 | 0.04 |
| Trop. ozone (eco) | kg NOx | 2.61 | 0.17 | 0.02 | 7.86 | 0.42 | 0 | 0.04 | 1.43E-03 | 0.05 |
| Terrestrial acidification | kg SO_2_ | 84.78 | 0.24 | 0.02 | 18.28 | 1.21 | 0 | 0.12 | 0.08 | 0.11 |
| Freshwater eutrophication | kg P | 0.46 | 0.03 | 9.82E-04 | 0.77 | 0.14 | 0 | 0.01 | 3.02E-04 | 1.51E-03 |
| Terrestrial ecotoxicity | kg 1,4-DCB | 4613.12 | 303.03 | 8.72 | 13191.95 | 900.20 | 0 | 60.16 | 5.46 | 17.10 |
| Freshwater ecotoxicity | kg 1,4-DCB | 325.36 | 2.41 | 0.08 | 102.74 | 8.76 | 0 | 0.68 | 0.02 | 0.12 |
| Marine ecotoxicity | kg 1,4-DCB | 62.64 | 3.44 | 0.12 | 144.47 | 12.42 | 0 | 0.71 | 0.03 | 0.21 |
| Human toxicity (cancer) | kg 1,4-DCB | 0.37 | 8.12 | 0.11 | 80.27 | 7.24 | 0 | 0.59 | 0.03 | 0.27 |
| Human toxicity (non-cancer) | kg 1,4-DCB | 286.17 | 72.15 | 2.57 | 3473.06 | 301.64 | 0 | 15.97 | 1.03 | 3.65 |
| Land use | m2a crop | 0 | 1.40 | 0.06 | 551.18 | 26.94 | 0 | 0.31 | 2.15 | 0.15 |
| Mineral resource | kg Cu | 0 | 1.62 | 0.01 | 22.71 | 5.11 | 0 | 0.43 | 2.44E-03 | 0.02 |
| Fossil resources | kg oil | 0 | 14.75 | 3.19 | 546.02 | 44.08 | 0 | 5.49 | 4.73 | 29 |
| Water use | m^3^ | 0 | 0.40 | 0.03 | 81.73 | 5.48 | 0 | 0.06 | 0.15 | 0.14 |

| **Table S5**  Midpoints data of ReCiPe2016 for producing 10 t of watermelon in Guilan province of Iran based on the breakdown of emissions factors. | | | | | | | | | | | | |
| --- | --- | --- | --- | --- | --- | --- | --- | --- | --- | --- | --- | --- |
| Midpoint | Unit | Emissions factors | | | | | | | | | | |
|  |  | On-Orchard emissions | Agricultural machinery | Lubricating oil | Nitrogen | Phosphate | Potassium | FYM | Pesticides | Fungicides | Diesel fuel | Electricity |
| Global warming | kg CO_2_ | 1091.75 | 112.45 | 3.51 | 1898.43 | 98.90 | 91.32 | 0 | 6.87 | 8.60 | 10.92 | 81.45 |
| Stratos. ozone depletion | kg CFC11 | 0.03 | 3.26E-05 | 2.72E-06 | 0.04 | 1.36E-04 | 7.07E-04 | 0 | 2.58E-05 | 4.66E-06 | 1.81E-05 | 4.23E-05 |
| Ionizing radiation | kBq Co-60 | 0 | 5.17 | 0.22 | 86.56 | 9.44 | 11.86 | 0 | 0.48 | 0.73 | 0.85 | 1.79 |
| Trop. ozone formation (hum) | kg NOx | 1.73 | 0.25 | 0.02 | 3.58 | 0.30 | 0.19 | 0 | 0.02 | 5.24E-04 | 0.04 | 0.15 |
| Particulate matter | kg PM2.5 | 5.04 | 0.22 | 7.53E-03 | 2.24 | 0.37 | 0.17 | 0 | 0.02 | 0.01 | 0.03 | 0.09 |
| Trop. ozone (eco) | kg NOx | 1.73 | 0.28 | 0.02 | 3.63 | 0.31 | 0.20 | 0 | 0.02 | 6.19E-04 | 0.04 | 0.15 |
| Terrestrial acidification | kg SO_2_ | 39.03 | 0.39 | 0.02 | 8.44 | 0.88 | 0.71 | 0 | 0.05 | 0.04 | 0.09 | 0.26 |
| Freshwater eutrophication | kg P | 0.35 | 0.05 | 1.09E-03 | 0.35 | 0.10 | 0.02 | 0 | 3.99E-03 | 1.31E-04 | 1.29E-03 | 4.01E-03 |
| Terrestrial ecotoxicity | kg 1,4-DCB | 2043.45 | 490.10 | 9.69 | 6090.69 | 657.03 | 230.34 | 0 | 26.31 | 2.36 | 14.61 | 166.48 |
| Freshwater ecotoxicity | kg 1,4-DCB | 138.09 | 3.90 | 0.09 | 47.43 | 6.39 | 2.05 | 0 | 0.30 | 0.01 | 0.10 | 2.65 |
| Marine ecotoxicity | kg 1,4-DCB | 27.67 | 5.57 | 0.13 | 66.70 | 9.06 | 2.36 | 0 | 0.31 | 0.01 | 0.18 | 3.37 |
| Human toxicity (cancer) | kg 1,4-DCB | 0.21 | 13.14 | 0.12 | 37.06 | 5.29 | 2.03 | 0 | 0.26 | 0.01 | 0.23 | 0.76 |
| Human toxicity (non-cancer) | kg 1,4-DCB | 146.10 | 116.68 | 2.85 | 1603.51 | 220.16 | 55.41 | 0 | 6.98 | 0.45 | 3.11 | 18.23 |
| Land use | m2a crop | 0 | 2.26 | 0.06 | 254.48 | 19.67 | 70.89 | 0 | 0.14 | 0.93 | 0.13 | 0.14 |
| Mineral resource | kg Cu | 0 | 2.62 | 0.01 | 10.48 | 3.73 | 0.28 | 0 | 0.19 | 1.06E-03 | 0.02 | 0.05 |
| Fossil resources | kg oil | 0 | 23.86 | 3.55 | 252.10 | 32.17 | 11.89 | 0 | 2.40 | 2.05 | 24.77 | 28.28 |
| Water use | m^3^ | 0 | 0.65 | 0.04 | 37.74 | 4 | 5.64 | 0 | 0.03 | 0.06 | 0.12 | 0.16 |
