# Supplementary material for: Data supporting midpoint-weighting life cycle assessment and energy forms of cumulative exergy demand for horticultural crops
Source: Data Brief. 2020 Nov 4;33:106490. doi: 10.1016/j.dib.2020.106490 (PMC7658572; doi:10.1016/j.dib.2020.106490)
Supplement: Supplementary file 3 [file mmc3.docx]

**Figure S1.** Share of each input to midpoints of citrus production.

**Figure S2.** Share of each input to midpoints of hazelnut production.

**Figure S3.** Share of each input to midpoints of kiwifruit production.

**Figure S4.** Share of each input to midpoints of tea production.

**Figure S5.** Share of each input to midpoints of watermelon production.
