# Supplementary material for: Data supporting midpoint-weighting life cycle assessment and energy forms of cumulative exergy demand for horticultural crops
Source: Data Brief. 2020 Nov 4;33:106490. doi: 10.1016/j.dib.2020.106490 (PMC7658572; doi:10.1016/j.dib.2020.106490)
Supplement: Supplementary file 5 [file mmc5.docx]

**Figure S6.** Weighting analysis of endpoints for citrus production.

**Figure S7.** Weighting analysis of endpoints for hazelnut production.

**Figure S8.** Weighting analysis of endpoints for kiwifruit production.

**Figure S9.** Weighting analysis of endpoints for tea production.

**Figure S10.** Weighting analysis of endpoints for watermelon production.
